# VSX2 and ASCL1 Are Indicators of Neurogenic Competence in Human Retinal Progenitor Cultures

**DOI:** 10.1371/journal.pone.0135830

**Published:** 2015-08-20

**Authors:** Lynda S. Wright, Isabel Pinilla, Jishnu Saha, Joshua M. Clermont, Jessica S. Lien, Katarzyna D. Borys, Elizabeth E. Capowski, M. Joseph Phillips, David M. Gamm

**Affiliations:** 1 Waisman Center, University of Wisconsin, Madison, Wisconsin, United States of America; 2 McPherson Eye Research Institute, University of Wisconsin, Madison, Wisconsin, United States of America; 3 Department of Ophthalmology, Lozano Blesa University Hospital, Zaragoza, Spain; 4 Aragones Health Sciences Institute, Zaragoza, Spain; 5 New England College of Optometry, Boston, Massachusetts, United States of America; 6 Department of Ophthalmology and Visual Sciences, University of Wisconsin, Madison, Wisconsin, United States of America; University of Michigan, UNITED STATES

## Abstract

Three dimensional (3D) culture techniques are frequently used for CNS tissue modeling and organoid production, including generation of retina-like tissues. A proposed advantage of these 3D systems is their potential to more closely approximate *in vivo* cellular microenvironments, which could translate into improved manufacture and/or maintenance of neuronal populations. Visual System Homeobox 2 (VSX2) labels all multipotent retinal progenitor cells (RPCs) and is known to play important roles in retinal development. In contrast, the proneural transcription factor Acheate scute-like 1 (ASCL1) is expressed transiently in a subset of RPCs, but is required for the production of most retinal neurons. Therefore, we asked whether the presence of VSX2 and ASCL1 could gauge neurogenic potential in 3D retinal cultures derived from human prenatal tissue or ES cells (hESCs). Short term prenatal 3D retinal cultures displayed multiple characteristics of human RPCs (hRPCs) found *in situ*, including robust expression of VSX2. Upon initiation of hRPC differentiation, there was a small increase in co-labeling of VSX2+ cells with ASCL1, along with a modest increase in the number of PKCα+ neurons. However, 3D prenatal retinal cultures lost expression of VSX2 and ASCL1 over time while concurrently becoming refractory to neuronal differentiation. Conversely, 3D optic vesicles derived from hESCs (hESC-OVs) maintained a robust VSX2+ hRPC population that could spontaneously co-express ASCL1 and generate photoreceptors and other retinal neurons for an extended period of time. These results show that VSX2 and ASCL1 can serve as markers for neurogenic potential in cultured hRPCs. Furthermore, unlike hESC-OVs, maintenance of 3D structure does not independently convey an advantage in the culture of prenatal hRPCs, further illustrating differences in the survival and differentiation requirements of hRPCs extracted from native tissue vs. those generated entirely *in vitro*.

## Introduction

Stem and progenitor cell proliferation and differentiation are controlled by complex inter- and intracellular interactions that direct the precise spatiotemporal production of particular cell types. The retina provides an excellent system to examine these developmental processes since it contains a limited number of major cell types arranged in a relatively simple neuronal network. The competence model of retinal cell fate determination has been put forth as an explanation for the conserved cell birth order observed during vertebrate retinal development [1,2]. An important premise of this model is the existence of a common retinal progenitor cell (RPC) that serves as the sole source for the six major neuronal cell types, as well as the Müller glia cells, that together constitute the neurosensory retina. The competency of an individual RPC to produce particular progeny is governed principally by instructive intrinsic cues controlled by a hierarchy of proneural transcription factors. These interactions result in a conserved but overlapping temporal order of differentiation and fate restriction that has been described in all examined vertebrate species: first, ganglion cells, followed by horizontal cells, cones, amacrine cells, rods, and then bipolar cells and Müller glia [3]. In addition to these temporal events, there is a layer of spatial complexity that exists during retinal development wherein differentiation begins in the central retina and proceeds towards the periphery. As such, at any single point during retinal development, the RPC pool is made up of a heterogeneous population with varying competencies [4–6].

The selection of appropriate markers is crucial for identifying and monitoring *bona fide* human RPCs (hRPCs) *in vitro*. Whereas there are many general indicators of proliferative neural progenitors in vertebrates (*eg*.,Ki67, Sox2, Ccnd1), the homeodomain transcription factor Vsx2 (Visual system homeobox 2, also known as Chx10) [7–9] is comparatively exclusive to the retina. Vsx2 is involved in maintenance and proliferation of the RPC pool, timing of photoreceptor production, and differentiation of one type of retinal interneuron, the bipolar cell [10–13]. In humans, *VSX2* mutations result in very small, nonfunctional eyes with correspondingly malformed retinas [14,15]. Affected individuals typically have a purely ocular phenotype, demonstrating the restricted tissue expression of VSX2 and the secondary effects of its dysfunction on global eye development [16]. VSX2 has also been used *in vitro* to identify multipotent RPCs derived from human ES cells (hESCs) and induced pluripotent stem cells (hiPSCs) [17–22]. Indeed, hiPSC-derived optic vesicle-like structures (OVs) from a patient with microphthalmia due to a mutation in the *VSX2* gene demonstrated defects in proliferation, enhanced retinal pigmented epithelial (RPE) cell differentiation at the expense of neural retina, and absence of bipolar cells [23]. These features are similar to those described for mutant *Vsx2* mouse models [10,11,24]. Thus, available evidence not only points toward Vsx2 being an essential element of RPCs in animal models, but in humans as well.

Another valuable marker used to identify progenitor cells is the proneural basic helix-loop-helix transcription factor Acheate scute-like 1 (Ascl1, also known as Mash1). Ascl1 has been shown to directly regulate the expression of genes involved in proliferation in the developing forebrain [25], and also to mark proliferating cells in the subventricular zone in human neocortex at midgestation [26] and in the adult brain [27]. In the mouse retina, Ascl1 is transiently expressed in RPCs and is required to generate all neural retinal cell lineages with the possible exception of ganglion cells [28,29]. This profound capacity to promote neural differentiation was illustrated in late passage cultures of glia-restricted RPCs and Mϋller glia, where ectopic ASCL1 expression was sufficient to restore neuronal potential [30,31]. However, despite its importance in retinal neurogenesis, co-expression of ASCL1 with VSX2 in hRPCs has not been examined to date.

The culture of RPCs from a human source is vital to the success of cell replacement therapies for retinal degenerative disease, and intense study is underway to apply developmental principles to understand and manipulate competency of hRPCs so as to produce sufficient quantities of desired cell types (*e*.*g*., photoreceptors). For this purpose, prenatal tissue seems to have a theoretical advantage since it harbors a large population of multipotent hRPCs produced in their natural milieu. Indeed, several reports have shown that prenatal hRPCs can undergo limited expansion in dissociated monolayer cultures [32–36]. An alternate strategy modeled after human cerebral cortical progenitor cultures was applied to prenatal retinal tissue whereby hRPCs were grown as 3D neurospheres and passaged by mechanical sectioning [37–39]. This approach eliminates the need for enzymatic digestion and maintains integral endogenous tissue structure and cell-cell contacts, and preserves cell surface receptors. Using this technique, hRPC cultures could be expanded for up to a year [30]. However, in humans and other mammals, the ability of RPCs and cortical progenitors to definitively generate new neurons declines with time as they become increasingly gliogenic, a phenomenon observed both in culture and *in vivo* [40–45].

The advent of embryonic stem cell (ESC) technology has provided another approach for the derivation of retinal cells, and numerous methods have been developed to generate all of the major retinal cell types in a time frame and sequence that mirror normal development [18,19,21]. In a landmark study, self-organizing neuroepithelium derived from mouse ESCs was shown to form 3D structures that resemble optic cups to a high degree and exhibit interkinetic nuclear migration and retinal lamination [46]. 3D optic vesicle-like structures from hESCs (hESC-OVs) have also been described, which can form multi-layered tissues with an inner layer of BRN3+ ganglion-like cells, an intermediate layer containing interneurons, and an outer layer of developing photoreceptor cells [20,24,47–50]. Beyond its value for the study of retinal development, it is conceivable that the *de novo* formation of a 3D structure that spatially approximates normal retinal tissue may be important for the appropriate maturation and function of resident retinal cells. Consistent with this notion, post-mitotic photoreceptors isolated from 2D monolayer mouse ESC retinal cultures demonstrated poor integration following subretinal transplantation into *Rho* ^*-/-*^, *Gucy2e* ^*-/-*^, and *Gnat1* ^*-/-*^ models of retinal degeneration [51]. However, when photoreceptor precursors from 3D mouse ESC-derived retinal cultures were transplanted into the same models, there was improved integration with outer segment maturation and establishment of synaptic connectivity [52]. Furthermore, reports have shown that 3D OVs from hESCs or hiPSCs can give rise to photoreceptors with advanced cellular architecture and functional capacity, including the ability to respond to light [47,50]. These studies indicate that the recapitulation of a 3D structural niche may play a beneficial role in photoreceptor generation and/or maturation.

Given the potential importance of neural retinal products for future therapeutic applications, we sought to determine the utility of VSX2 and ASCL1 to serve as predictors of multipotent neurogenic potential in 3D prenatal hRPC neurosphere cultures and 3D OV cultures derived from human pluripotent stem cells (hPSCs). Here we show that VSX2 and ASCL1 co-expression does correlate with neurogenic competence in hRPC cultures, although their expression in prenatal retinal neurospheres is short-lived and does not ensure demonstration of multipotency. In contrast, VSX2+ hRPCs derived from hESC-OVs are capable of co-expressing ASCL1 and generating multiple types of retinal neurons, including photoreceptor lineage cells, over an extended period of time in culture.

## Materials and Methods

### Ethics Statement

The method of collection for human retinal tissue conformed to the NIH guidelines for the receipt of such tissues and adhered to the tenets of the Declaration of Helsinki. All donors to the University of Washington-Birth Defects Research Laboratory provided written informed consent for the collection and use of their samples for research purposes. Institutional Review Board approval was obtained from the University of Washington-Human Subjects Division and the University of Wisconsin Health Sciences Institutional Review Board.

### Tissue Collection and Cell Culture

Human prenatal tissue was obtained from the Laboratory of Developmental Biology at the University of Washington-Seattle. Neural retina and RPE cultures were prepared from individual postmortem human prenatal eyes of gestational ages between 60 and 125 days, which were estimated using crown-to-rump and foot length measurements at the time of collection [39]. Briefly, after removal of the anterior portion of the eye cup and vitreous, the retina was carefully detached without disturbing the underlying RPE. Margins of anterior retina near the ora serrata and posterior retina surrounding the optic nerve were excluded from the dissection. The tissue was sectioned into 200 μm cubes with a McIlwain tissue chopper, seeded into T75 flasks, and cultured as neurospheres in standard medium consisting of DMEM/HAMS F12 (3:1), 1% antibiotic-antimycotic (penicillin-streptomycin- amphotericin), 2% B27 (Life Technologies, Carlsbad, CA), 20 ng/ml EGF (Sigma-Aldrich, St. Louis MO), 20 ng/ml FGF2 (R&D Systems, Minneapolis, MN), and 5 μg/ml heparin (Sigma-Aldrich, St. Louis MO). Alternatively, neurospheres were cultured in standard medium conditioned for 24 hr by prenatal human RPE monolayers (RPE CM) [30]. For all cultures, half of the medium was replenished every 1 to 2 days. Of note, short term neurospheres were not passaged; however, long term neurospheres (i.e., cultured for 2 months) were passaged once via mechanical sectioning as described previously [39].

### Human Embryonic Stem Cell Culture and Retinal Differentiation

hESC culture and retinal differentiation were performed as previously described [21]. Briefly, the hESC line WA09 (obtained from WiCell, Madison, WI) was maintained on an irradiated mouse embryonic fibroblast feeder layer in hESC medium (DMEM:F12 1:1; 20% knockout serum; 1% MEM non-essential amino acids; 1% L-glutamine; β-mercaptoethanol; 20 ng/ml FGF2). Retinal differentiation was initiated by lifting embryoid bodies (EBs) with 2 mg/ml dispase (Life Technologies, Carlsbad, CA) and culturing them as free-floating EBs in hESC media without FGF2 for 3 days. On day 4, cultures were switched to Neural Induction Medium (DMEM:F12; 1% N2 supplement (Life Technologies, Carsbad, CA), 1% MEM non-essential amino acids; 1% L-glutamine and 2 mg/ml heparin) and plated onto a laminin-coated surface at day 7. At day 16, the loosely adherent neural clusters were lifted from the plate by mechanical trituration, switched to Retinal Differentiation Medium (DMEM:F12 3:1; 2% B27 without retinoic acid and 1% anti-mycotic/antibiotic). At day 20, optic vesicle-like structures (OVs) were manually selected based their distinct phase bright, neuroepithelial appearance under light microscopy and maintained in Retinal Differentiation Medium. For a subset of experiments, the WA01 line (obtained from WiCell) was cultured and differentiated as described for the WA09 line. At the conclusion of all hESC studies, short tandem repeat testing was performed (WiCell) to confirm the identities of the WA09 and WA01 hESC lines (data not shown).

### RPE Conditioned Medium (RPE CM) Production

RPE from human prenatal eyes was seeded on flasks coated with 10 μg/ml laminin (Sigma Aldrich, St. Louis, MO) and cultured as monolayers in standard medium as previously described [53]. Conditioned medium from the RPE cultures was collected daily, sterile-filtered through a 0.2 μm membrane, and supplemented with 2% B27, 20 ng/ml EGF, 20 ng/ml FGF2, and 5 μg/ml heparin before use [30].

### DAPT Treatment

For Notch inhibition studies, prenatal retinal neurospheres were dissociated to single cell suspensions by addition of Accutase (Millipore, Temecula CA) for 10 min at 37°C. Cells (50,000 in 50 μl) were plated onto laminin/poly-L-ornithine-coated glass coverslips in standard medium for 24 hr, treated with either vehicle (0.2% dimethyl sulfoxide) (Sigma-Aldrich, St. Louis MO) or 10 μM N-[N-(3,5-difluorophenacetyl)-L-alanyl]-S phenylglycine t-butyl ester (DAPT) (Sigma-Aldrich, St. Louis MO) in standard medium for 24 hr, washed 3 times to remove DAPT, and cultured for an additional 7 days in medium supplemented with 2% B27 only.

### Immunohistochemistry

Upon receipt of tissue, some eyes were prepared for immunohistochemical analysis by fixation with 4% paraformaldehyde in PBS for 1 hr, cryoprotection with 15% sucrose for 1 hr and 30% sucrose overnight at 4°C, OCT imbedding, and cryostat sectioning (15 μm) [54]. Eye sections were incubated with primary antibodies overnight at room temperature. Labeled cells were visualized with either Alexa 488- or Cy3-conjugated secondary antibodies and nuclei were counterstained with 4’6’diamidino-2-phenylindole (DAPI). Serial confocal images were collected with an A1 laser scanning fluorescence confocal microscope using EZ-C1 software (Nikon Corp., Tokyo Japan). OVs derived from hESCs at 20, 50, or 90 days of differentiation were fixed, sectioned, and immunostained as described previously [22].

### Immunocytochemistry

Prenatal retinal neurospheres or hESC-derived OVs were dissociated into single cell suspensions by addition of Accutase for 10 min at 37°C and 50,000 cells (1000 cells/μl) were plated onto laminin/poly-L-ornithine-coated glass coverslips for 24 hr in standard medium (acute dissociation) or 7 days in standard medium without mitogens to initiate differentiation. Cells were then fixed with 4% paraformaldehyde in PBS and incubated with primary antibodies for either 2 hr at room temperature or overnight at 4°C. Labeled cells were visualized via fluorescence microscopy with either Alexa 488- or Cy3-conjugated secondary antibodies and nuclei were counterstained with DAPI.

### Cell counts

Cell counts were performed using a Nikon fluorescence microscope (40X objective) and Nikon Elements Imaging software. Quantification was performed by counting the total number of DAPI-stained nuclei and the number of cells immunostained with selected markers. At least 3 coverslips and 6 independent fields/coverslip were used for counting, with each field containing a minimum of 100 cells (total area > 25 mm^2^).

### RT-PCR and quantitative RT-PCR (qRT-PCR)

RNA isolation and cDNA synthesis were performed as previously described [53]. The cDNA templates were diluted 1:40 and added to PCR reactions containing GoTaq Master Mix (Promega, Madison WI) and 10 μM each of the appropriate forward and reverse primers (S1 Table). Samples were initially denatured for 5 min at 95°C followed by 30 cycles of PCR amplification (95°C for 15 sec, 60°C for 30 sec, 72°C for 1 min) and a final extension for 10 min at 72°C. PCR products were visualized on a 1.5% agarose gel containing 0.1% ethidium bromide. PCR reactions were repeated using at least three different prenatal retinal neurosphere cultures to ensure reproducibility. RT-qPCR (40 cycles) was also performed as described using primer pairs that spanned at least one intron, SYBR Green 2X PCR Master Mix (Applied Biosystems, Foster City CA), and the ABI 7500 PCR System (Applied Biosystems, Foster City, CA) [53].

### Antibody sources

Anti-ASCL1 (MASH1) (1:100, mouse monoclonal, BD Pharmingen, Franklin Lakes, NJ); anti-CHX10 (VSX2) (1:200 goat polyclonal, Santa Cruz Biotechnology, Santa Cruz CA or 1:250 sheep polyclonal, Exalpha Biologicals, Shirley MA); anti-KI67 (1:500 mouse monoclonal, BD Pharmingen, Franklin Lakes, NJ); anti-NESTIN (1:200 rabbit polyclonal, Millipore Temecula CA); anti-PKCα (1:100 rabbit polyclonal, Santa Cruz Biotechnology, Santa Cruz CA); anti-RECOVERIN (1:2000 rabbit polyclonal, Millipore, Temecula CA): anti-SOX2 (1:2000 goat polyclonal or 1:100 mouse monoclonal, R and D Systems, Minneapolis MN); anti-ßIII TUBULIN (1:5000 mouse monoclonal, Sigma-Aldrich, St. Louis MO or 1:5000 rabbit polyclonal, Covance, Princeton NJ). All antibodies were validated in previous studies [21,23,30].

### Statistics

Statistical analyses were performed using Prism software version 3.02 for Windows (GraphPad Software, San Diego, CA). Results were expressed as mean ± standard error (SEM), and significance was determined using Student’s paired *t* tests for cell count data and Student’s unpaired *t* tests for qRT-PCR data. All experiments were repeated using 3 or more independent cell cultures.

## Results

### VSX2 and KI67 expression identify a proliferating retinal progenitor population in prenatal human eye and 3D retinal neurospheres

**Fig 1:**
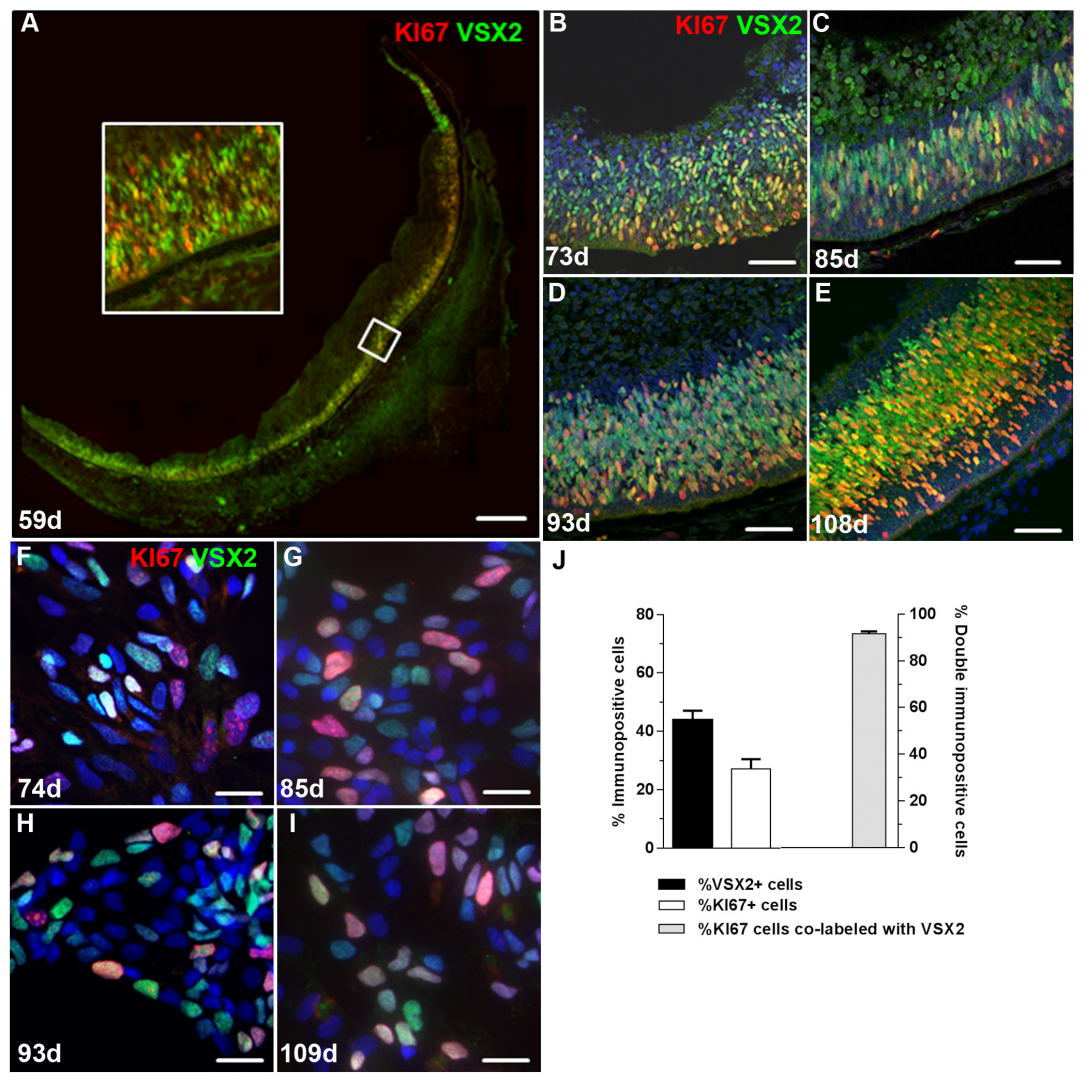
Short term cultures of human retinal neurospheres retain a robust population of VSX2+ proliferating progenitor cells from source prenatal retinal tissue. VSX2+/KI67+ proliferating hRPCs were observed in the outer neuroblastic layer of the developing retina at (**A**) 59 days, (**B**) 73 days, (**C**) 85 days, (**D**) 93 days, and (**E**) 108 days of gestation. (**F-I**) VSX2+/KI67+ co-labeled cells were also present in dissociated cells from short term prenatal retinal neurospheres established from retinal tissue of similar gestational ages. (**J**) Short term prenatal retinal neurospheres were dissociated and immunostained to determine the percentage of cells expressing VSX2 and/or KI67. Nuclei were visualized with DAPI. The insert is a 4X magnification of the indicated area in panel A. Scale bars: 100 μm (panel A); 50 μm (panels B-E); 20 μm (panels F-I).

Early light microscopic and ultrastructure studies initially established the existence of a mitotic cell in the outer neuroblastic layer of the developing human retina [55–57]. The cell proliferation marker KI67 has subsequently been used to identify these mitotic progenitor cells as early as the 6th week of gestation and extending past the 23rd week [33,58–60]. In corroboration of such studies, we observed a robust population of KI67+ cells in the outer neuroblastic layer of human retina from 59 to 108 days of gestation (S1 Fig). To characterize these proliferating cells in greater detail, cryosections were prepared from post-mortem prenatal human eyes, immunolabeled with KI67, and counterstained with the retina-specific progenitor marker VSX2 (Fig 1A–1E). Abundant VSX2 immunolabeling was detected throughout the outer neuroblastic layer and retinal periphery in day 59 prenatal eye (Fig 1A). The vast majority of KI67+ cells was associated with this VSX2+ population at day 59 and throughout the rest of the time points examined (Fig 1B–1E).

To determine if hRPCs maintained expression of these markers *in vitro* when cultured as 3D retinal neurospheres, tissue was isolated from age-matched prenatal retina from 74 to 109 days of gestation, mechanically sectioned into 200 μm cubes, and grown as free-floating neurosphere cultures in defined medium supplemented with the mitogens EGF and FGF2 [53]. After 1 week *in vitro*, neurospheres were dissociated into single cells which were subsequently plated onto coverslips and analyzed using immunocytochemistry (Fig 1F–1J, S2 Fig). Similar to our observations *in situ*, each 3D retinal neurosphere culture was initially comprised of large populations of KI67+ proliferating cells (27.2 ± 3.3% of total cells) and VSX2+ cells (44.0 ± 3.4%), and nearly all KI67 immunolabel was associated with VSX2+ cells (91.6 ± 1.0%).

**Fig 2:**
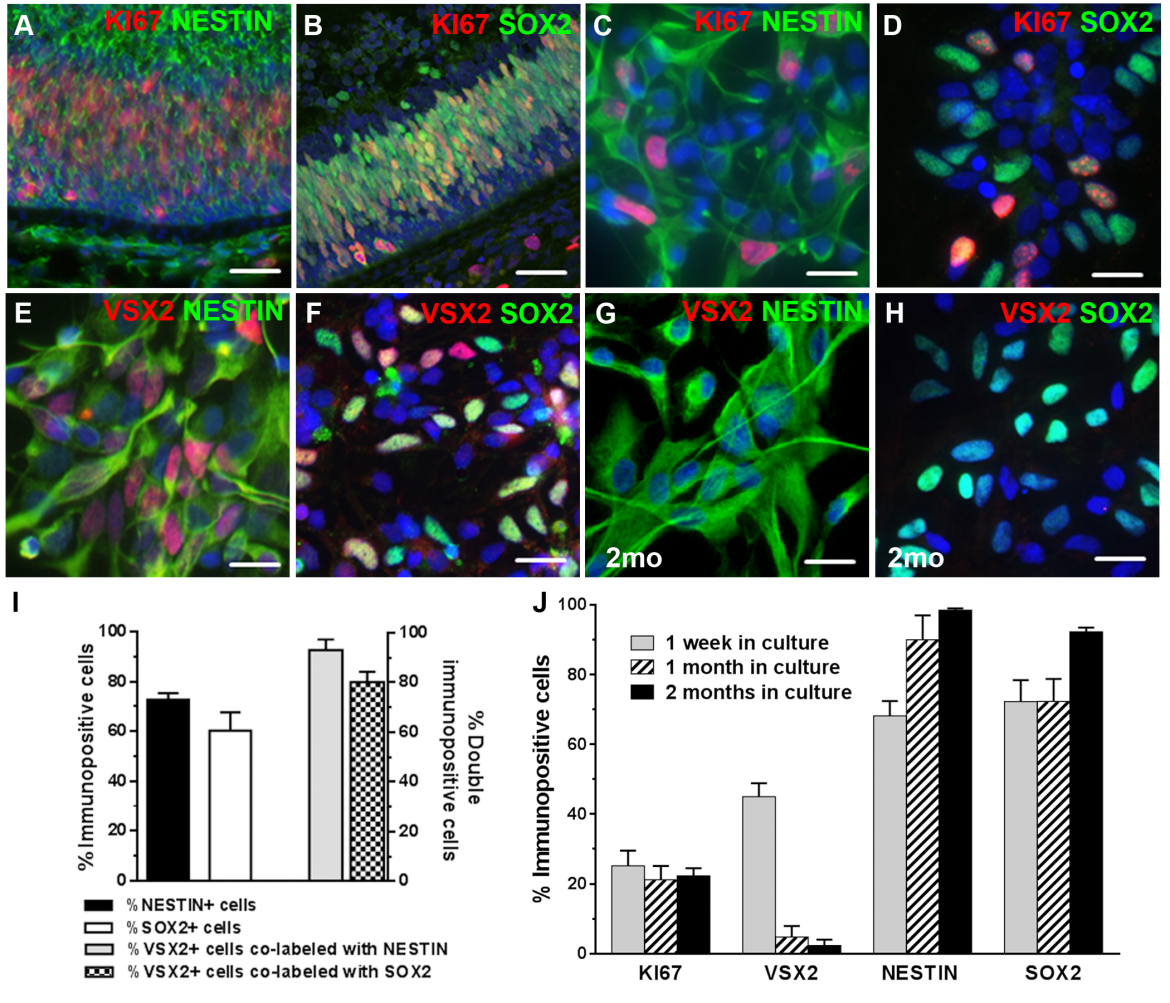
Prenatal retinal neurospheres lose VSX2 expression over time in culture. KI67+ hRPCs in the outer neuroblastic layer of 96 day human prenatal retina co-express the neural stem cell markers (**A**) NESTIN and (**B**) SOX2. Short term prenatal retinal neurosphere cultures also contain abundant (**C**) KI67+/NESTIN+ and (**D**) KI67+/SOX2+ hRPCs. Nearly all VSX2+ hRPCs in short term prenatal retinal neurosphere cultures co-label with (**E**) NESTIN and (**F**) SOX2. Prenatal retinal neurosphere cultures (n = 5) from 79–108 day gestation tissue were sampled at 1 week, 1 month, and 2 months. After 2 months, very little VSX2 immunostaining is detected, although (**G**) NESTIN and (**H**) SOX2 remain highly expressed. The percentage of VSX2, KI67, NESTIN, and SOX2 immunopositive cells were quantified (**I**) in short term cultures and (**J**) over a 2 month period. Nuclei were visualized with DAPI and cell count data is expressed as % immunopositive cells. Scale bars: 50 μm (panels A,B); 20 μm (panels C-H).

In prenatal tissue, KI67+ cells were also highly associated with NESTIN (Fig 2A) and SOX2 (Fig 2B), which are both general markers for neural progenitor populations. When examined in 3D prenatal neurospheres after one week in culture, these markers were expressed in a large proportion of the total plated cells (NESTIN: 72.7 ± 2.7%; SOX2: 60.3 ± 7.4%) (Fig 2C, 2D and 2I). When the VSX2+ population was analyzed for co-expression of these markers, the overwhelming majority of VSX2+ cells co-labeled with NESTIN (93.0 ± 4.3%) (Fig 2E and 2I) and SOX2 (80.1 ± 4.2%) (Fig 2F and 2I). These results show that 3D prenatal retinal neurosphere cultures initially contain a substantial percentage of proliferating cells that nearly uniformly co-express VSX2 and other RPC markers.

We then sought to determine whether the retinal developmental program initiated *in vivo* could be continued in 3D prenatal neurosphere cultures *in vitro*. hRPC cultures (n = 5) were established and grown for 2 months in standard medium supplemented with RPE conditioned medium to support continued growth and survival and more closely mimic the *in vivo* environment [30]. Neurospheres from each culture were collected at 1 week, 1 month, and 2 months *in vitro*, dissociated into single cell suspensions, plated on coverslips, and either acutely fixed for KI67 immunocytochemistry or withdrawn from mitogens for 7 days to initiate differentiation, followed by immunocytochemical analysis for RPC markers. The number of cells expressing VSX2 declined to <3% during the two month time period (Fig 2G, 2H and 2J); however, nearly all cells remained immunopositive for NESTIN (Fig 2G and 2J) and SOX2 (Fig 2H and 2J) at 2 months *in vitro*. Furthermore, KI67 immunolabeling remained unchanged over this two month period, indicating that cell proliferation proceeded unabated in culture despite the striking decline in VSX2 expression (Fig 2J).

### Neurogenesis in hRPCs decreases over time in 3D neurosphere cultures

**Fig 3:**
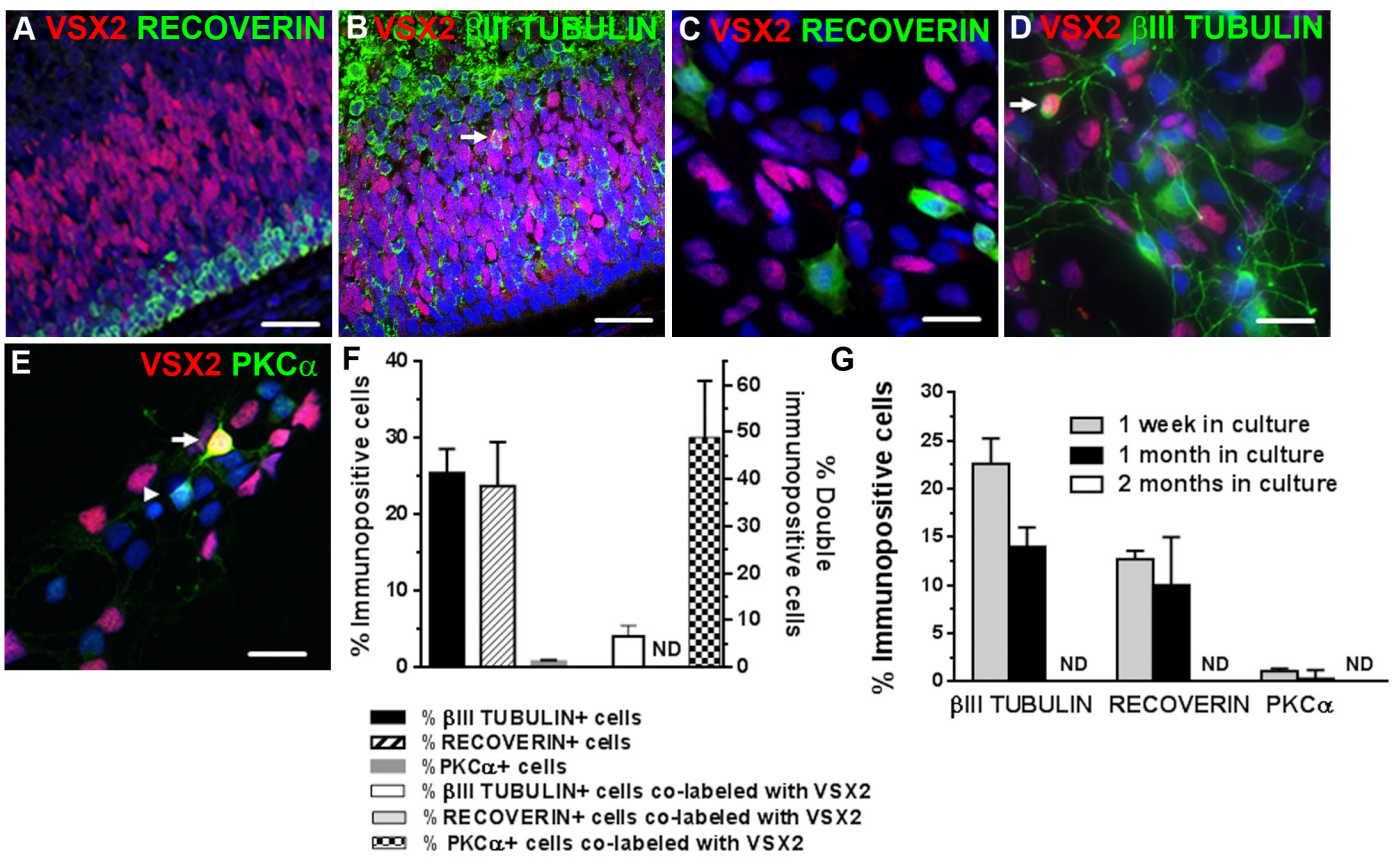
Neurogenesis decreases over time in human prenatal retinal neurosphere cultures. VSX2 immunoreactivity is not associated with (**A**) RECOVERIN+ cells in prenatal retinal tissue but does co-label a small subset of (**B**) βIII TUBULIN+ neurons. A similar pattern of VSX2 co-expression with (**C**) RECOVERIN and (**D**) βIII TUBULIN is observed in short term prenatal retinal neurosphere cultures; *arrow* in panels B and D indicate VSX+/βIII TUBULIN+ cells. (**E**) A subpopulation of PKCα+ cells also co-labels with VXS2 (*arrow* and *arrowhead* in panel E indicate VSX+/PKCα+ and VSX2-/PKCα+ cells, respectively). (**F**) The percentage of cells expressing VSX2 and selected neuronal markers were quantified in short term cultures. (**G**) Human prenatal retinal neurosphere cultures (n = 5) from day 79–108 gestation tissue were sampled at 1 week, 1 month, and 2 months, and the number of cells immunolabeled with selected neuronal markers was quantified. Nuclei were visualized with DAPI and cell count data is expressed as % immunopositive cells. ND: nondetectable. Scale bars: 50 μm (panels A,B); 20 μm (panels C-E).

Expression of RECOVERIN, a marker for photoreceptors and a subset of cone bipolar cells [61], and ßIII TUBULIN, a general marker of post-mitotic neurons [62], was abundant in day 90 primary prenatal retinal tissue (Fig 3A and 3B), and both RECOVERIN+ (23.7 ± 5.8%) and ßIII TUBULIN+ cells (25.5 ± 3.1%) (Fig 3C, 3D and 3F) were found in dissociated 3D retinal neurosphere cultures after one week *in vitro*. No RECOVERIN+/VSX2+ double-labeled cells were seen, but rare ßIII TUBULIN+/VSX2+ neurons were observed in day 93 prenatal retina sections and in dissociated primary cultures (arrowheads in Fig 3B and 3D).

VSX2 also serves as a marker for a subset of bipolar cells, which are late-born, post-mitotic retinal interneurons [63]. To determine the percentage of post-mitotic VSX2+ bipolar cells in prenatal 3D retinal neurospheres, dissociated cultures were co-immunostained for VSX2 and PKCα, a marker for rod bipolar cells and some cone bipolar cells [61,64] (Fig 3E). Additional cultures were co-labeled with PKCα and ßIII TUBULIN, which revealed that all PKCα+ cells were ßIII TUBULIN+ as well (S3 Fig). When the percentages of cells expressing PKCα and/or VSX2 were quantified, few PKCα+ bipolar cells were detected (0.82 ± 0.21%) (Fig 3F). However, in this rare PKCα+ population, nearly 50% of the cells co-expressed VSX2 (Fig 3F). Since all PKCα+ cells were also ßIII TUBULIN+ (S3 Fig), the ßIII TUBULIN+/VSX2+ double-labeled cells were likely bipolar cells. Altogether, these results show that the overwhelming majority of VSX2+ cells in early prenatal retinal neurospheres are hRPCs derived from the outer neuroblastic layer *in situ*, with only a minute contribution from postmitotic VSX2+ bipolar cells.

To examine the fate of neuronal and photoreceptor populations in 3D retinal neurospheres over time, we performed immunocytochemistry after 1 week, 1 month, and 2 months *in vitro*. ßIII TUBULIN and RECOVERIN expression decreased dramatically at 1 month (14.0 ± 2.0% and 10.0 ± 5.0%, respectively), and both markers were undetectable by 2 months (Fig 3G). Likewise, no PKCα immunopositive cells were detected at ≥1 month *in vitro* (Fig 3G). Thus, neither the production nor the maintenance of neuronal populations was supported in long term cultures of 3D prenatal retinal neurospheres, consistent with our previously published results [30].

### VSX2+ prenatal hRPCs cultured short term as 3D neurospheres express ASCL1 upon mitogen withdrawal

**Fig 4:**
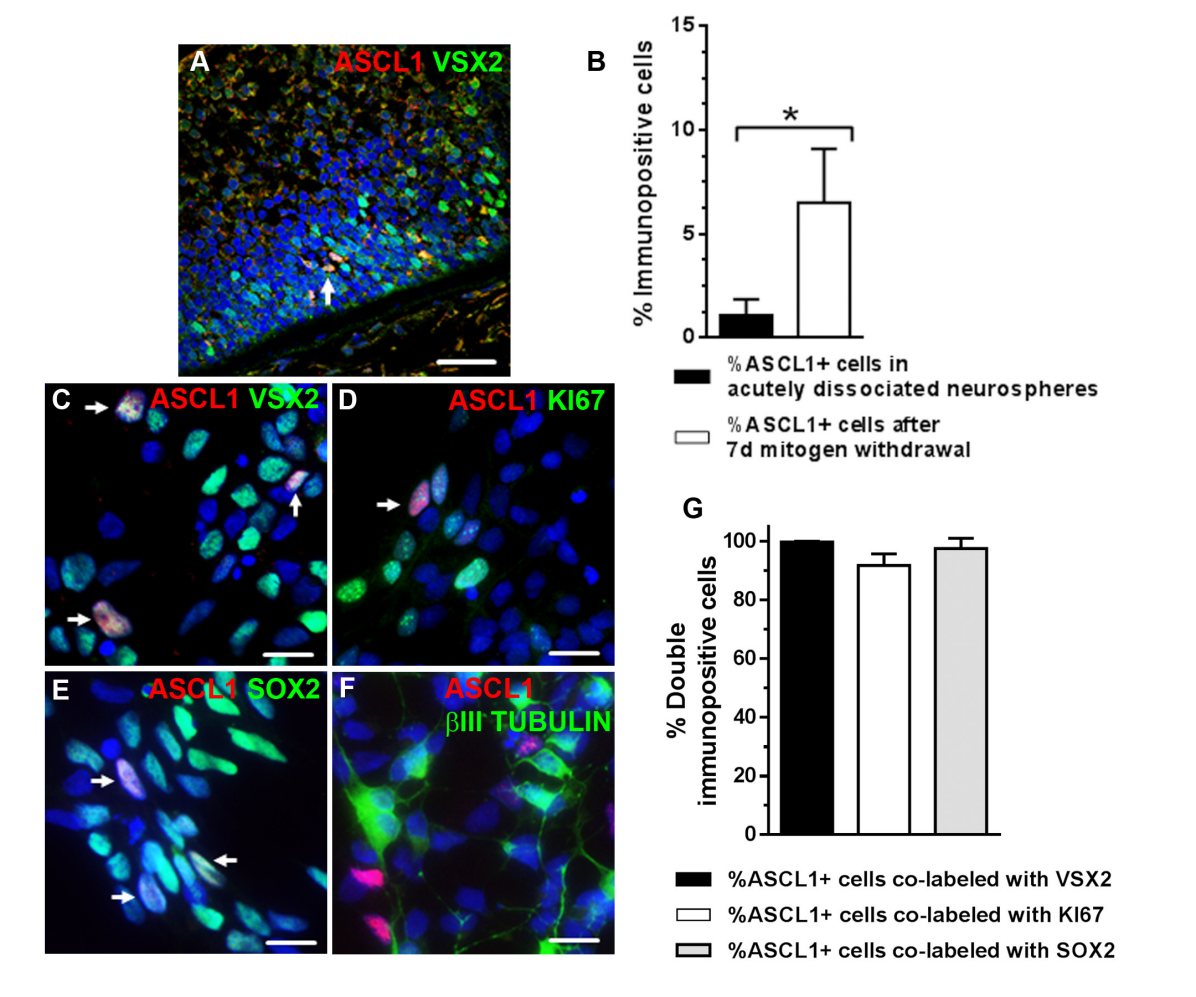
VSX2-positive hRPCs from short term cultures of human prenatal retinal neurospheres express ASCL1 upon differentiation. (**A**) VSX2+/ASCL1+ hRPCs were present in 90 day gestation human prenatal retinal tissue. (**B**) The percentage of ASCL1+ cells in short term prenatal human retinal neurosphere cultures was determined by immunocytochemistry after mitogen withdrawal. ASCL1 immunoreactivity co-localized with (**C**) VSX2, (**D**) KI67, and (**E**) SOX2, but not with (**F**) ßIII TUBULIN. (**G**) The percentages of ASCL1+ cells that co-labeled with individual progenitor markers after differentiation was determined. Nuclei were visualized with DAPI. *p<0.05. *Arrows* in panels A and C-E designate ASCL1+ nuclei. Scale bar: 50 μm (panel A); 20 μm (panels C-F).

The proneural transcription regulator, Ascl1, plays an essential role in neurogenesis throughout the mouse central nervous system and is required for the differentiation of specific retinal neurons [29,65,66]. When we examined day 90 prenatal retinal sections for ASCL1 expression, we observed that ASCL1 immunolabel was detected exclusively in VSX2+ cells (Fig 4A). In short term (1 week) 3D retinal neurosphere cultures, we observed an increase in ASCL1 expression after mitogen withdrawal (3.46 ± 0.57-fold, p = 0.001), as well as increased expression of the ASCL1 gene targets HES6 (1.79 ± 0.28-fold, p = 0.015) and DLL1 (2.10 ± 0.41, p = 0.036) (S4A Fig). Other progenitor and neurogenic markers were also quantified in short term 3D prenatal retinal neurospheres. No significant differences were seen in VSX2, KI67, NESTIN, or SOX2 expression after mitogen withdrawal when compared to cultures examined prior to removal of mitogens (data not shown). However, there was a significant increase in the percentage of cells expressing the proneural transcription factor ASCL1 (6.05 ± 2.59% vs. 1.09 ± 0.78%, p = 0.024; Fig 4B). Of note, essentially all ASCL1+ cells were immunopositive for VSX2 (99.7 ± 0.3%; Fig 4C and 4G), KI67 (91.8 ± 4.0%; Fig 4D and 4G), and SOX2 (97.5 ± 3.5%; Fig 4E and 4G). In contrast, no co-labeling of ASCL1 with the post-mitotic neuronal markers ßIII TUBULIN (Fig 4F) or RECOVERIN (data not shown) was detected before or after initiation of neurogenesis by mitogen withdrawal. In addition, since all PKCα+ cells were immunopositive for ßIII TUBULIN in these cultures, we could infer that ASCL1 was not co-expressed with PKCα. Therefore, prenatal VSX2+ hRPCs in 3D retinal neurospheres could initiate neurogenesis, as demonstrated by co-expression of ASCL1, but production of discrete neural phenotypes was not observed under these culture conditions.

### The neurogenic potential of VSX2+ hRPCs in 3D prenatal retinal neurospheres is modestly augmented following inhibition of NOTCH signaling

**Fig 5:**
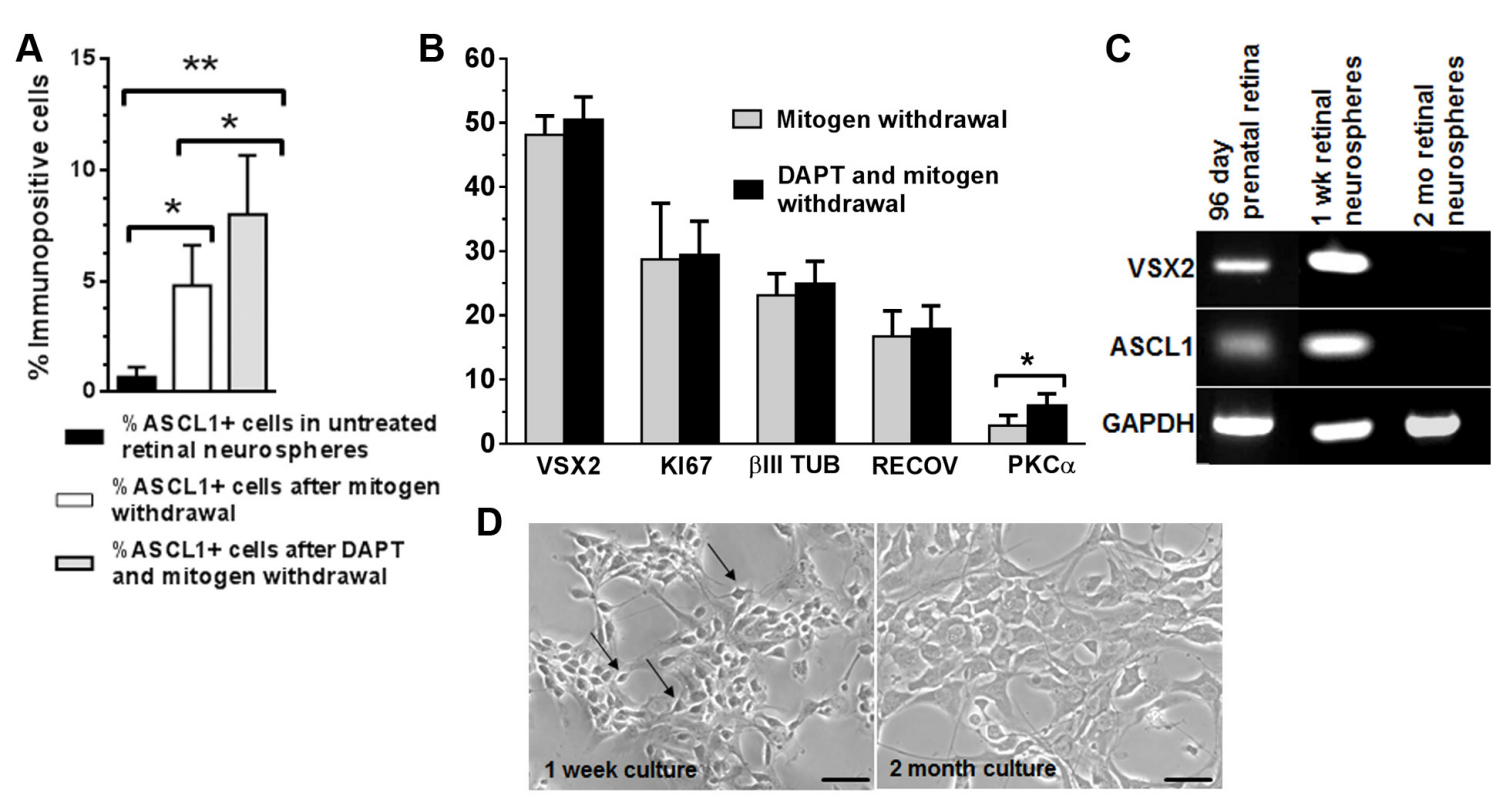
NOTCH inhibition augments production of PKCα+ neurons in short term prenatal human retinal neurospheres. The percentages of cells immunopositive for (**A**) ASCL1 and (**B**) other selected hRPC and neuronal markers following DAPT treatment was determined by immunocytochemistry. (**C**) RT-PCR analysis was used to evaluate expression of VSX2 and ASCL1 in prenatal human retinal tissue and short (1 week) and long (2 month) term prenatal retinal neurosphere cultures. (**D**) Phase photomicrographs of dissociated prenatal retinal neurospheres reveal profound cell morphology differences between short term and long term cultures. Cell counts are expressed as % immunopositive cells. *p<0.05; **p<0.01. Scale bars in panel E: 50 μm.

Notch signaling plays a crucial role in RPC maintenance and glial cell differentiation *in vivo* and *in vitro* [67]. In the canonical Notch pathway, members of the Jag or Dll families of ligands bind to the Notch receptor, resulting in release of the Notch intracellular domain (NICD) and activation of a number of direct target genes, including Hes1 and Hes5. These basic helix-loop-helix proteins serve as potent repressors of several proneural transcription regulators, including Ascl1. To determine whether inhibition of NOTCH signaling could enhance neurogenesis in 3D prenatal retinal neurospheres, cultures were treated after 1 week *in vitro* with the γ-secretase inhibitor DAPT (10 μM) or vehicle for 24 hr after mitogen withdrawal and subjected to RT-qPCR and immunocytochemical analyses (Fig 5A). The expression level of ASCL1 increased in the DAPT-treated group compared to untreated controls (1.49 ± 0.16-fold, p = 0.038), as did expression of the ASCL1 gene regulatory targets HES6 (1.68 ± 0.25-fold, (p = 0.027) and DLL1 (1.65 ± 0.06-fold, p = 0.01) (S4B Fig).

Consistent with the RT-qPCR data, DAPT treatment increased the total percentage of ASCL1 immunolabeled cells compared to vehicle (8.01 ± 2.21% vs. 4.81 ± 1.82%, p = 0.035) (Fig 5A). No significant changes were seen in the total percentages of cells expressing VSX2, KI67, ßIII TUBULIN, or RECOVERIN following DAPT treatment (Fig 5B). As observed previously, ASCL1+ cells co-localized with VSX2 (99.7 ± 0.33%) and KI67 (93.3 ± 0.7%), but not with ßIII TUBULIN or RECOVERIN (data not shown). Interestingly, the number of PKCα+ neurons increased with DAPT treatment (5.97 ± 1.86% vs. 2.86 ± 1.59%, p = 0.017) (Fig 5B, S5 Fig). A similar effect was seen in mouse retinal explants following overexpression of *Ascl1* in proliferating Müller glia [31]. Thus, in short term cultures of 3D prenatal retinal neurospheres, hRPCs maintained the potential to produce at least one neuronal cell type and remained responsive to changes in NOTCH signaling.

After 2 months, VSX2 and ASCL1 expression was lost in prenatal 3D retinal neurosphere cultures (Fig 5C). All other neurogenic marker immunoreactivity was also undetectable in dissociated or intact retinal neurospheres, whereas NESTIN and SOX2 were expressed in the vast majority of the long term culture cell population (shown in Fig 2G, 2H and 2J). Unlike short term cultures, DAPT treatment of long term prenatal retinal neurosphere cultures had no effect on the expression of ASCL1, VSX2, PKCα, or other neuronal markers (data not shown). A striking change in cell morphology was also observed by 2 months *in vitro*, at which point the entire culture was composed of large, flat cells with broad processes, as opposed to the phase bright neuronal cells seen after short term culture (Fig 5D). These results confirm that hRPCs grown as 3D prenatal retinal neurospheres lose neurogenic potential in long term culture, and further demonstrate that VSX2 and ASCL1 co-expression in hRPCs correlates with maintenance of neurogenic potential.

### VSX2 and ASCL1 expression can be maintained long term in optic vesicle cultures derived from hESCs

**Fig 6:**
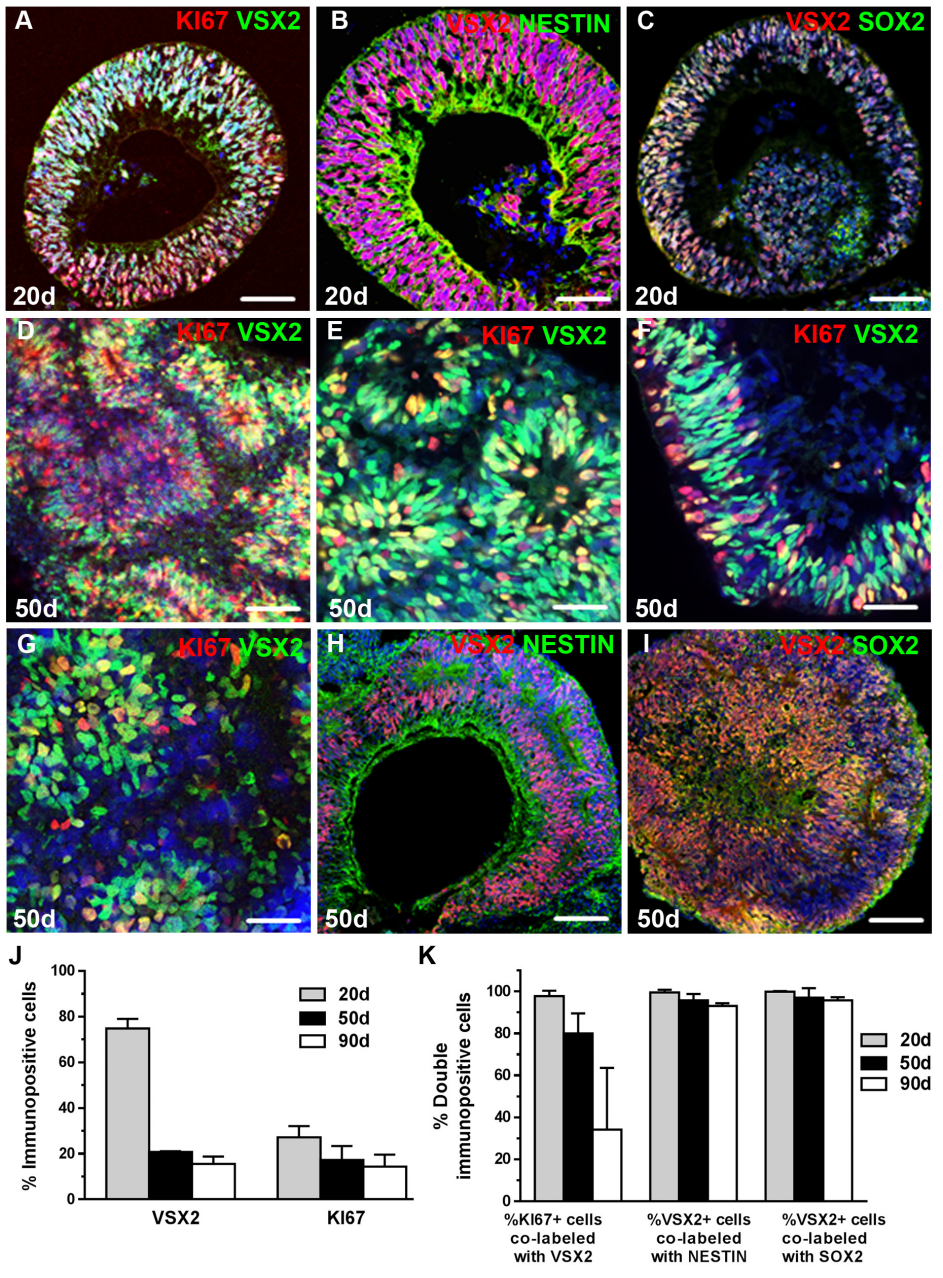
VSX2+ hRPCs are abundant within short and long term cultures of 3D optic vesicles derived from hESCs. 20 days after initiation of retinal differentiation [20], WA09 hESCs formed optic vesicle structures (OVs) comprised of VSX2+ hRPCs co-expressing (**A**) KI67, (**B**) NESTIN, and (**C**) SOX2. (**D-F**) At day 50, the majority of VSX2+ hRPCs remained KI67+. **(G)** VSX2+/KI67+ progenitors were also present at day 50 in hESC-OVs derived from the WA01 line. 50 day VSX2+ hRPCs continued to express (**H**) NESTIN and (**I**) SOX2. (**J**) At 20, 50, and 90 days of differentiation, the percentages of VSX2+ or KI67+ cells and (**K**) VSX2+ cells co-labeled with other progenitor markers were quantified. Nuclei were visualized with DAPI and cell count data is expressed as % immunopositive cells. Scale bar: 50 μm (panels A-D,H,I); 20 μm (panels E,F,G).

To further probe the capacity of VSX2 and ASCL1 co-expression to serve as an indicator of neurogenic competence *in vitro*, we examined 3D optic vesicle (OV) cultures derived from hESCs. The hESC line WA09 was directed toward a retinal fate using our established protocol [20,21], and hESC-OVs were isolated after 20 days of differentiation and maintained thereafter in suspension culture. At the 20 day time point, hESC-OVs were comprised of a nearly pure population of VSX2+ cells (Fig 6A–6C) that were also KI67+ (Fig 6A), NESTIN+ (Fig 6B), and SOX2+ (Fig 6C), similar to human prenatal retinal neurospheres. hESC-OVs exhibited vigorous cell proliferation, leading to an average increase in individual OV size of 800% from day 20 through day 70 of differentiation (S6 Fig). At 50 days of differentiation, robust KI67+/VSX2+ proliferating progenitor populations were often found in rosette-like formations (Fig 6D and 6E); however, a KI67+/VSX2+ stratum resembling the outer neuroblastic layer was maintained in some hESC-OVs (Fig 6F). Identical results were observed at 50 days of differentiation using a second hESC line, WA01 (Fig 6G). The progenitor status of 50 day VSX2+ cells was confirmed by co-labeling with NESTIN (Fig 6H) and SOX2 (Fig 6I), and both the rate of proliferation and the progenitor status of VSX2+ cells were maintained in 90 day cultures (Fig 6 and 6K). Of note, a decrease in hESC-OV VSX2 expression was observed from day 20 to day 50 owing to spontaneous hRPC differentiation.

**Fig 7:**
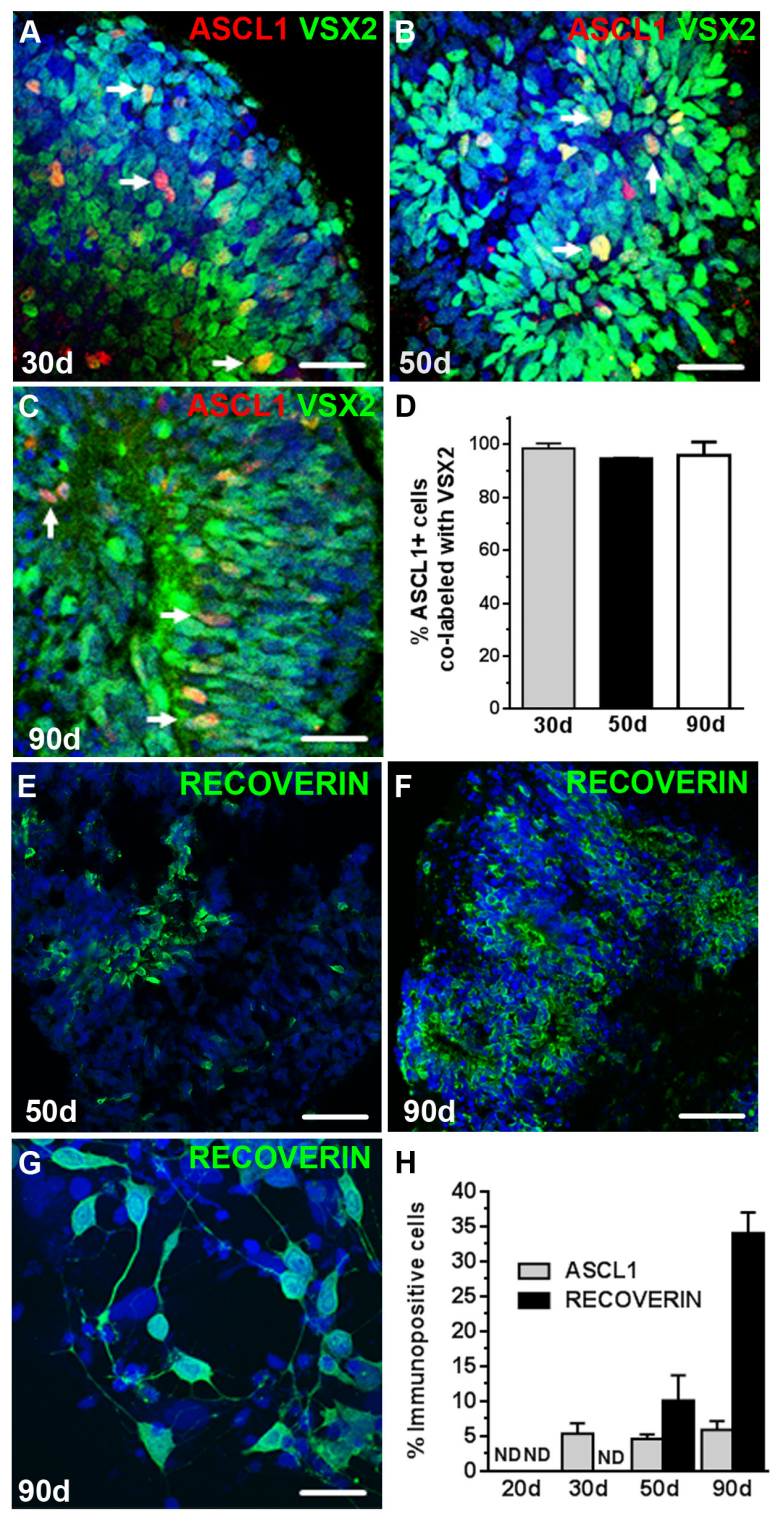
VSX2+ hRPCs from 3D hESC-OVs maintain neurogenic competence and continue along a normal developmental trajectory in long term cultures. VSX2+/ ASCL1+ hRPCs were detected in 3D hESC-OVs at (**A**) 30 days, (**B**) 50 days, and (**C**) 90 days of differentiation. (**D**) The percentages of ASCL1+ cells that co-labeled with VSX2 were quantified in 30, 50, and 90 day hESC-OVs. (**E**) By 50 days, many photoreceptor precursor cells identified by RECOVERIN immunoreactivity were present in hESC-OVs, and by 90 days (**F,G**), RECOVERIN+ cells increased in abundance. (**H**) Quantification of cells expressing ASCL1 or RECOVERIN was performed at 20, 30, 50, and 90 days of differentiation. Nuclei were visualized with DAPI and cell count data is expressed as % immunopositive cells. ND: nondetectable. *Arrows* in panels A-C demarcate ASCL1+/VSX2+ cells. Scale bars: 50 μm (panels E,F); 20 μm (panels A-C,); 10 μm (panel G).

No ASCL1+ cells were observed at day 20 in hESC-OVs, although a population of ASCL1+ progenitors (5.35% ± 1.47) was detected at 30 days of differentiation, which corresponds to the onset of neurogenesis in these cultures (Fig 7A). Unlike prenatal retinal neurosphere cultures, a stable percentage of ASCL1+ cells was observed over the entire time period tested (50 days: 4.60% ± 0.68, Fig 7B; 90 days: 5.90% ± 1.27, Fig 7C and 7H). At every time point, ASCL1 immunoreactivity was associated almost exclusively with VSX2+ progenitor cells (Fig 7D). As expected, no RECOVERIN+ photoreceptor precursors were seen at 30 days of differentiation, but at day 50, RECOVERIN+ cells made up a considerable portion of the culture (10.1% ± 3.6, Fig 7E and 7H). By day 90, the percentage of RECOVERIN+ cells in differentiating hESC-OVs increased even further (34.1% ± 3.0), consistent with normal human retinal neurogenesis (Fig 7F, 7G and 7H).

## Discussion

Recent studies using 3D culture systems have demonstrated that organogenesis can be modeled *in vitro*. Gut, liver, kidney, and CNS organoids have been successfully derived from ESCs, iPSCs, and embryonic tissues through a self-assembly process that is largely intrinsically driven (for review see [68–70]). It is theorized that mechanical, hydrodynamic, and structural influences create a dynamic environment that provides the necessary context for lineage restriction and spatial organization. Retinal differentiation has served as a key example of this concept, with both mouse and human pluripotent stem cells giving rise to highly mature, tissue-like structures via self-organizing OVs [20,24,47,49,52,71].

In the absence of influences from tissues surrounding the developing retina, juxtacrine communication through intercellular contact may play a primary role in hRPC differentiation *in vitro* [72]. Thus, we reasoned that maintenance of local physical structure of prenatal retina in culture would provide a more conducive environment for hRPCs to proceed along their normal differentiation program. To test this hypothesis, we employed a hRPC isolation protocol that preserved native cell-cell contacts and surface receptors, and avoided passaging except in the case of long term (2 month) cultures, which were passaged once using the same method. At first inspection, prenatal retinal neurospheres, like hESC-OVs, seemed to provide an environment that allowed resident hRPCs to maintain a normal developmental trajectory. Indeed, prenatal retinal neurospheres initially contained robust populations of proliferating VSX2+ cells that co-expressed SOX2 and NESTIN, suggesting multipotent hRPC status. However, unlike hESC-OVs, both the expression of VSX2 and the generation of new retinal neurons was not maintained in prenatal retinal neurospheres after short term culture.

To further evaluate the capacity to make new retinal neurons from cultured prenatal retinal neurospheres, we examined ASCL1 expression over time. ASCL1 plays a pivotal role in neuronal production [73,74], and its forced expression has been shown to directly convert mouse and human fibroblasts to functional neuronal cells [75], which it accomplishes by binding to a large number of neurogenic promoters and activating broad neuronal transcriptional programs [76]. ASCL1 is also involved in neural progenitor proliferation [25], and transient proliferating ASCL1+ progenitors have been identified in rodent and human embryonic and adult brain [26,27,77,78]. Proliferating progenitors expressing Ascl1 have also been shown to give rise to most major neuronal types in mouse retina, including photoreceptors [29]. In the present study, we describe for the first time the presence of a proliferating ASCL1+/VSX2+ progenitor in the outer neuroblastic area of prenatal human retina. VSX2 and ASCL1 co-expression was carried over into short term prenatal retinal neurosphere cultures, and upon differentiation, ASCL1 was upregulated exclusively in VSX2+ cells, consistent with the initiation of neurogenesis from the hRPC population. Treatment with the NOTCH inhibitor DAPT further increased ASCL1 expression in short term prenatal retinal neurospheres and augmented expression of ASCL1 target genes such as DLL1 and HES6, which are involved in the neuronal differentiation program [79–81]. However, although VSX2+ hRPCs appeared to retain neurogenic potential in prenatal retinal neurosphere cultures, only one type of retinal neuron, a PKCα+ bipolar cell, was found in greater abundance following DAPT treatment. Also, like VSX2, ASCL1 expression was completely lost over time, with no additional neurogenesis observed after 1 month in culture, either spontaneously or after DAPT treatment. In contrast to prenatal retinal neurospheres, 3D hESC-OVs maintained a proliferating VSX2+/ASCL1+ progenitor cell population and continued to produce an abundance of photoreceptor cells over an extended period of time without a requirement for exogenous NOTCH inhibition.

Although some 2D prenatal retinal culture systems have been shown to promote the survival and/or generation of primary neurons *in vitro* [32–35], other groups have reported the loss of neuronal production over time [30,41–43], similar to what is observed in human prenatal cortical neurosphere cultures [45,82]. These distinct findings likely reflect differences in culture methods and suggest that sudden removal of prenatal hRPCs from their native milieu makes them susceptible to diversion from their inherent differentiation program. Strategies designed to more closely simulate *in vivo* conditions may ultimately lead to improved prenatal hRPC survival and expansion in 2D or 3D cultures with retention of multipotent neurogenic potential [36] [30].

As opposed to prenatal hRPCs, retinal progenitors born and differentiated entirely from hPSC cultures spontaneously initiate and maintain a near-normal program of retinal development under minimal culture conditions. Consistent with this observation, hPSC-derived hRPCs, but not prenatal retinal neurospheres, contained a continuous subpopulation of cells that co-expressed VSX2 and ASCL1, indicative of persistent neurogenic competence. If hRPCs are to be employed as versatile tools for basic science and clinical applications, it is important to preserve their capacity both for long term propagation and neuronal (including photoreceptor) production. With these considerations in mind, it appears that pluripotent stem cells present a better option than prenatal hRPCs for large-scale production of the full cohort of retinal neurons for use in disease modeling and future cell replacement therapies.

## Supporting Information

S1 Fig Proliferating cells are predominantly localized to the outer neuroblastic layer of the developing human retina. KI67 immunolabeling was used to identify proliferating cells in tissue sections of (**A,B**) 59 day, (**C**) 76 day, (**D**) 85 day, (**E**) 90 day, and (**F**) 108 day human prenatal retinas. Panel A is a 10X magnification composite image of retinal sections from a 59 day donor eye, whereas panels B-F are 40X magnification images. The box in panel A designates the approximate retinal region where the images in panels B-F were obtained. Scale bar: 50 μm.
(TIF)

S2 Fig Short term cultures of human retinal neurospheres retain a robust population of VSX2+ proliferating progenitor cells from source prenatal retinal tissue. Composite micrographic images from Fig 1 depicting VSX2 and KI67 labeling were separated into single channel images to illustrate the abundance of proliferating VSX2+ cells in short term hRPC cultures. Scale bar: 20 μm.
(TIF)

S3 Fig PKCα+ cells constitute a subset of the βIII TUBULIN-expressing neuronal population in short term prenatal retinal neurosphere cultures. Prenatal retinal neurospheres were grown for 7 days, dissociated, and immunostained for PKCα (red) and βIII TUBULIN (green). Nuclei were counterstained with DAPI. The *arrow* indicates a PKCα+/βIII TUBULIN+ neuron. Scale bar: 20 μm.
(TIF)

S4 Fig *ASCL1*, *HES6*, and *DLL1* expression is increased following mitogen removal and DAPT treatment. Short term cultures of human prenatal retinal neurospheres were dissociated and (**A**) incubated in the absence of mitogens for 7 days to promote differentiation, followed by RT-qPCR analysis to quantify expression levels of ASCL1, HES6, and DLL1 relative to undifferentiated cultures, or (**B**) treated with the Notch inhibitor DAPT or vehicle for 24 hr and cultured for an additional 7 days without mitogens. RT-qPCR analysis then was used to quantify levels of expression of ASCL1, HES6, and DLL1 in DAPT- vs. vehicle-treated cultures. *p<0.05; **p<0.01.
(TIF)

S5 Fig Notch inhibition increases the number of bipolar cells in short term prenatal retinal neurosphere cultures. Prenatal retinal neurospheres were grown for 7 days, dissociated, treated with 10 μM DAPT for 24 hr, differentiated for an additional 7 days, and immunostained for VSX2 (red) and PKCα (green). Nuclei were counterstained with DAPI. *Arrows* and *arrowheads* indicate PKCα+/VSX2+ and PKCα+/VSX2- neurons, respectively. Scale bar: 20 μm.
(TIF)

S6 Fig Optic vesicle structures derived from human ESCs undergo sustained and robust proliferation for more than 70 days in culture. Individual day 20 hESC-OVs (n = 6) were isolated and grown for an additional 50 days in individual wells of a 96-well plate. (**A**) Phase-bright light microscopic images were taken every 5 days, followed by assessment of sphere area and percent increase in size over time. (**B**) Representative images of a single sphere over time. Scale bar: 100 μm.
(TIF)

S1 Table List of primers used for RT-PCR and qRT-PCR. (DOCX)
