# Supplementary figures and images for: VSX2 and ASCL1 Are Indicators of Neurogenic Competence in Human Retinal Progenitor Cultures
Source: PLoS One. 2015 Aug 20;10(8):e0135830. doi: 10.1371/journal.pone.0135830 (PMC4546156; doi:10.1371/journal.pone.0135830)

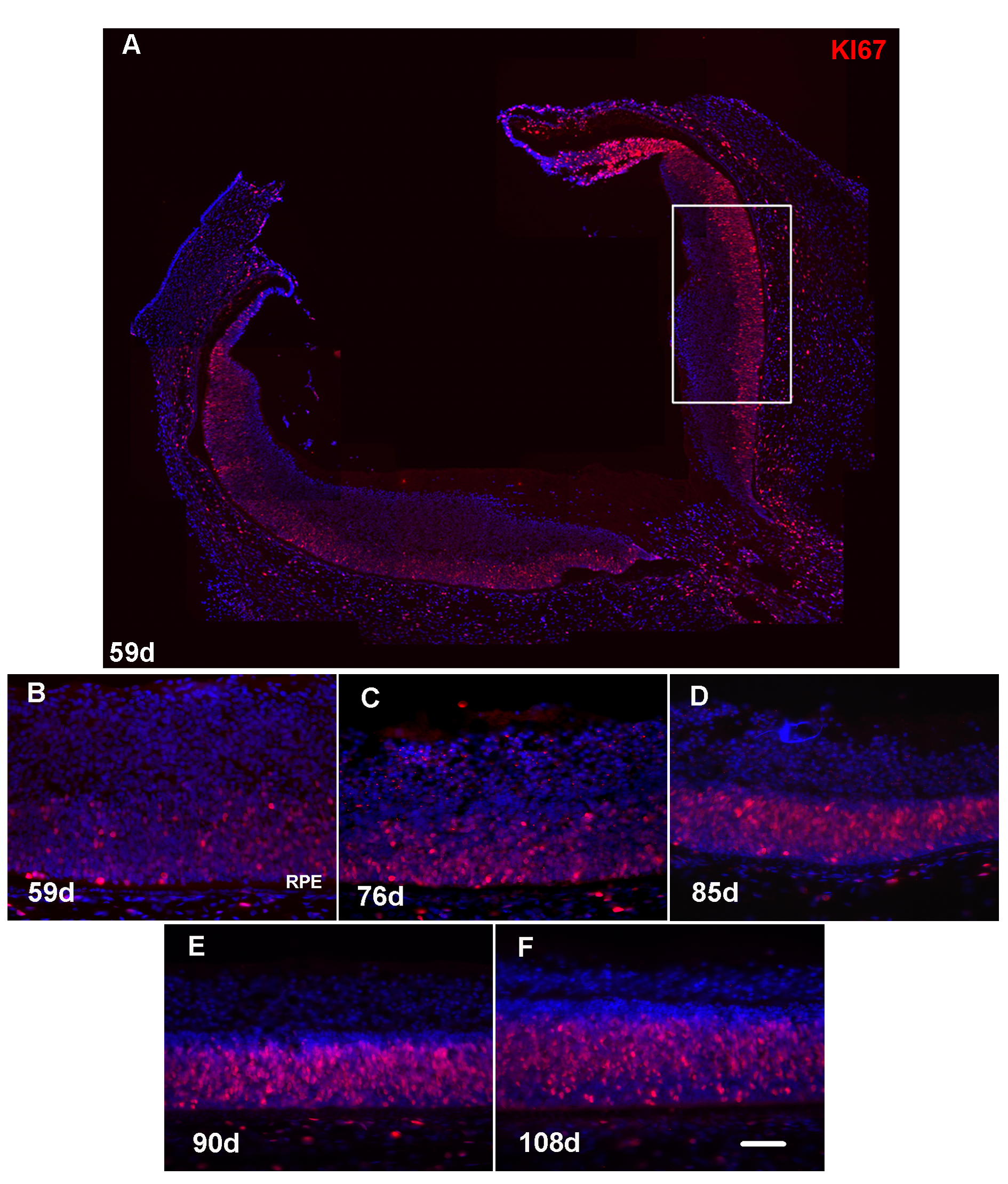

Supplement: S1 Fig — KI67 immunolabeling was used to identify proliferating cells in tissue sections of (A,B) 59 day, (C) 76 day, (D) 85 day, (E) 90 day, and (F) 108 day human prenatal retinas. Panel A is a 10X magnification composite image of retinal sections from a 59 day donor eye, whereas panels B-F are 40X magnification images. The box in panel A designates the approximate retinal region where the images in panels B-F were obtained. Scale bar: 50 μm. (TIF) [file pone.0135830.s001.tif]

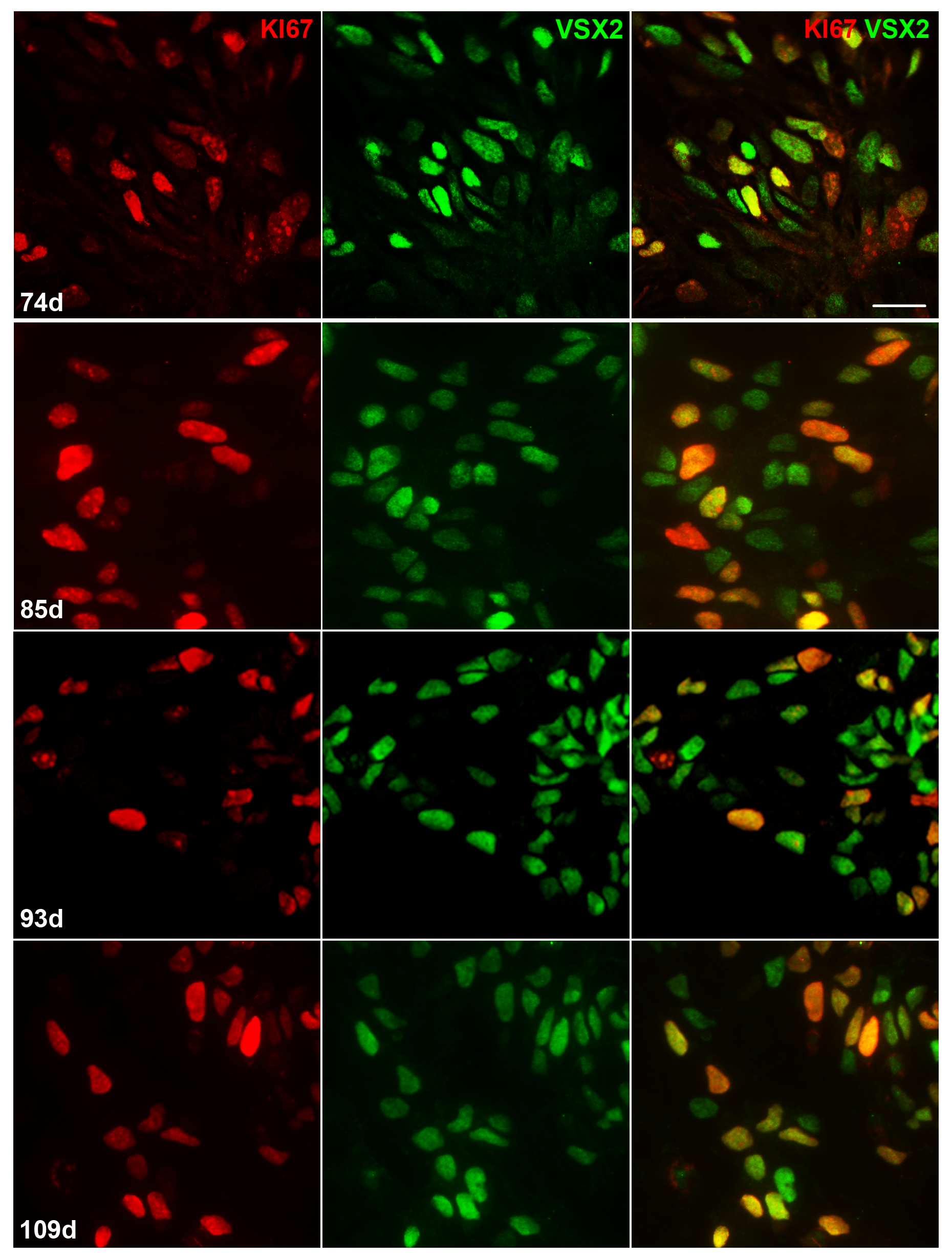

Supplement: S2 Fig — Composite micrographic images from Fig 1 depicting VSX2 and KI67 labeling were separated into single channel images to illustrate the abundance of proliferating VSX2+ cells in short term hRPC cultures. Scale bar: 20 μm. (TIF) [file pone.0135830.s002.tif]

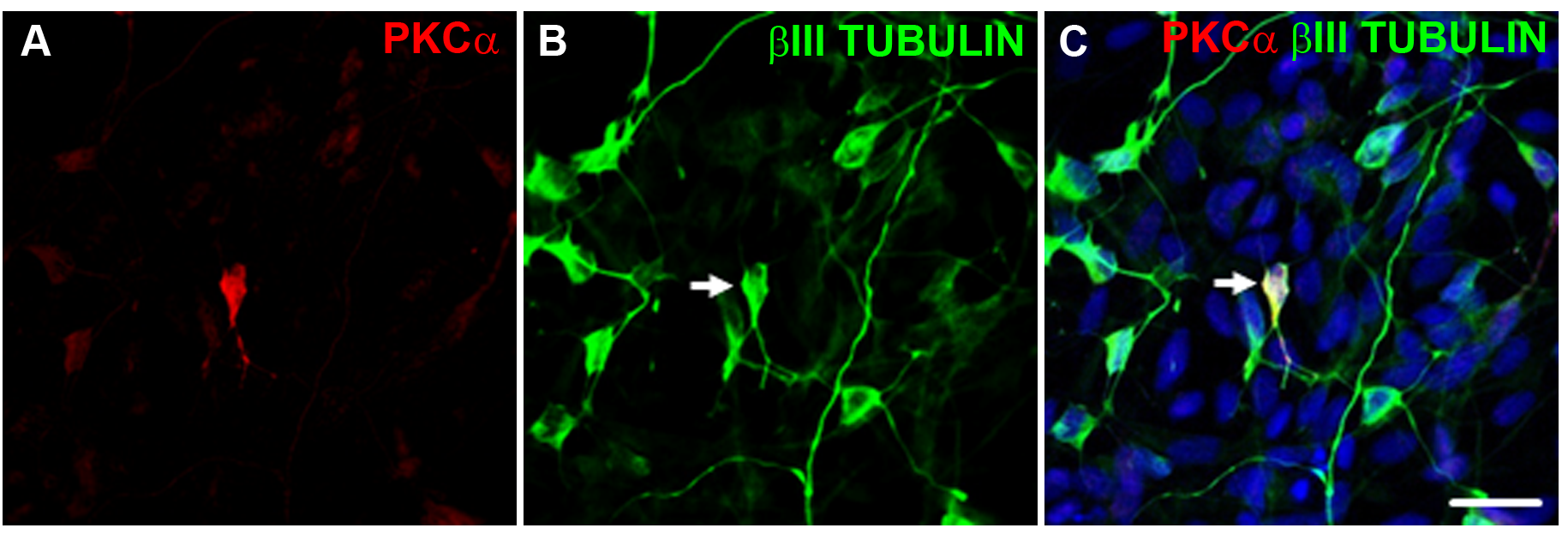

Supplement: S3 Fig — Prenatal retinal neurospheres were grown for 7 days, dissociated, and immunostained for PKCα (red) and βIII TUBULIN (green). Nuclei were counterstained with DAPI. The arrow indicates a PKCα+/βIII TUBULIN+ neuron. Scale bar: 20 μm. (TIF) [file pone.0135830.s003.tif]

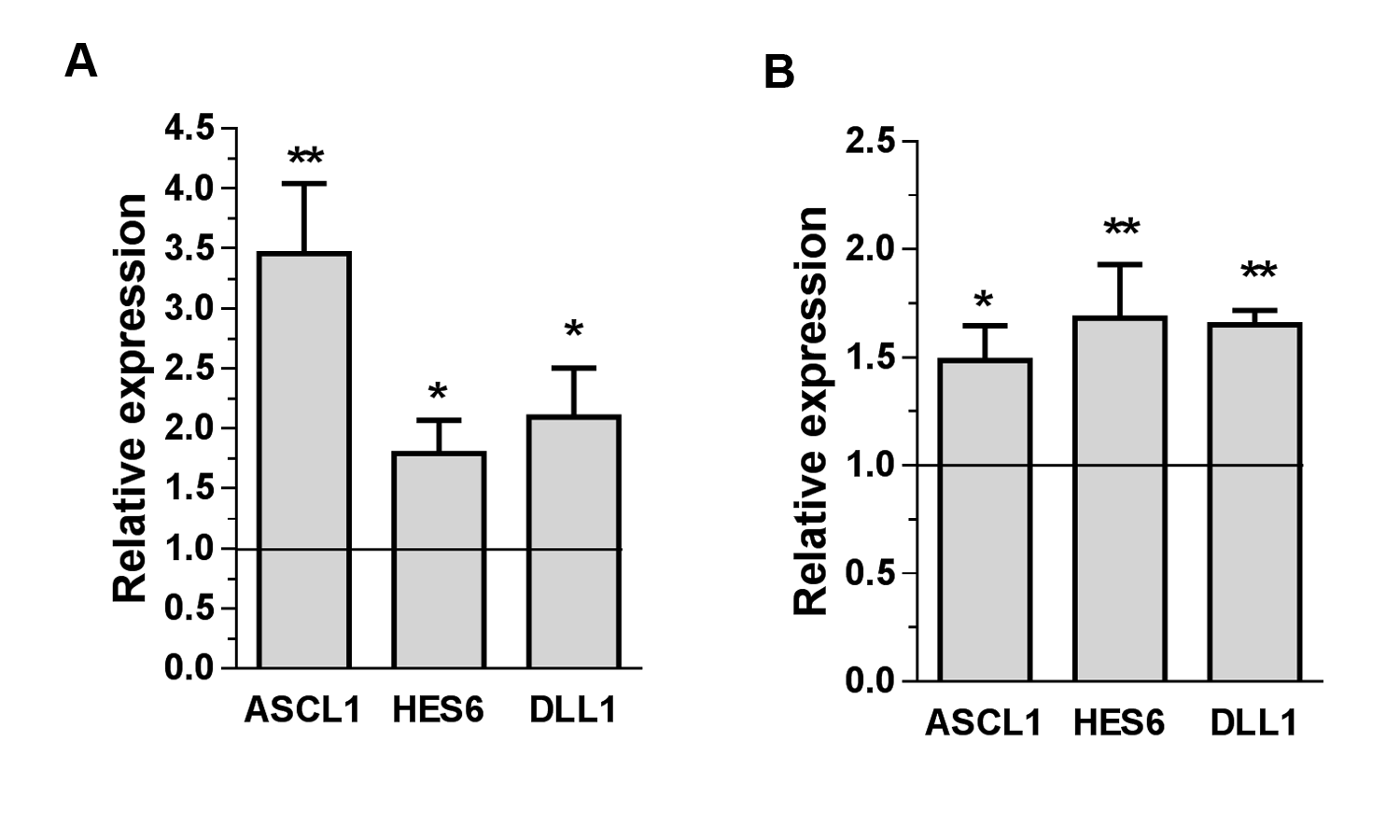

Supplement: S4 Fig — Short term cultures of human prenatal retinal neurospheres were dissociated and (A) incubated in the absence of mitogens for 7 days to promote differentiation, followed by RT-qPCR analysis to quantify expression levels of ASCL1, HES6, and DLL1 relative to undifferentiated cultures, or (B) treated with the Notch inhibitor DAPT or vehicle for 24 hr and cultured for an additional 7 days without mitogens. RT-qPCR analysis then was used to quantify levels of expression of ASCL1, HES6, and DLL1 in DAPT- vs. vehicle-treated cultures. *p<0.05; **p<0.01. (TIF) [file pone.0135830.s004.tif]

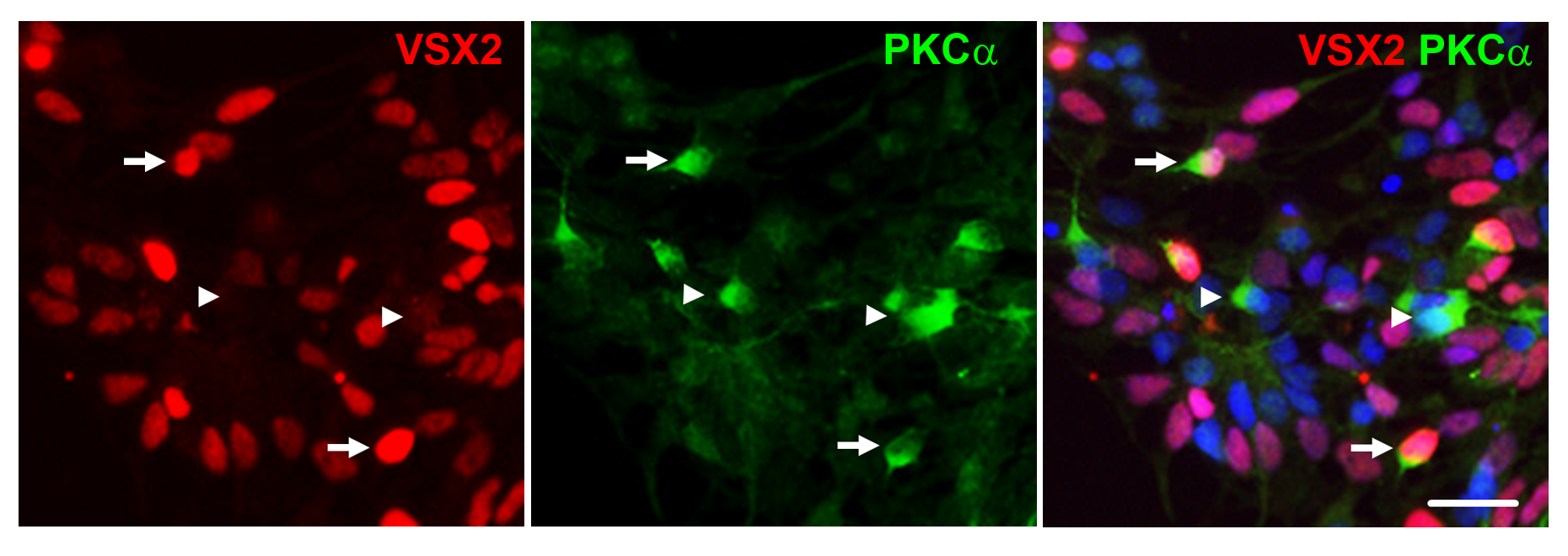

Supplement: S5 Fig — Prenatal retinal neurospheres were grown for 7 days, dissociated, treated with 10 μM DAPT for 24 hr, differentiated for an additional 7 days, and immunostained for VSX2 (red) and PKCα (green). Nuclei were counterstained with DAPI. Arrows and arrowheads indicate PKCα+/VSX2+ and PKCα+/VSX2- neurons, respectively. Scale bar: 20 μm. (TIF) [file pone.0135830.s005.tif]

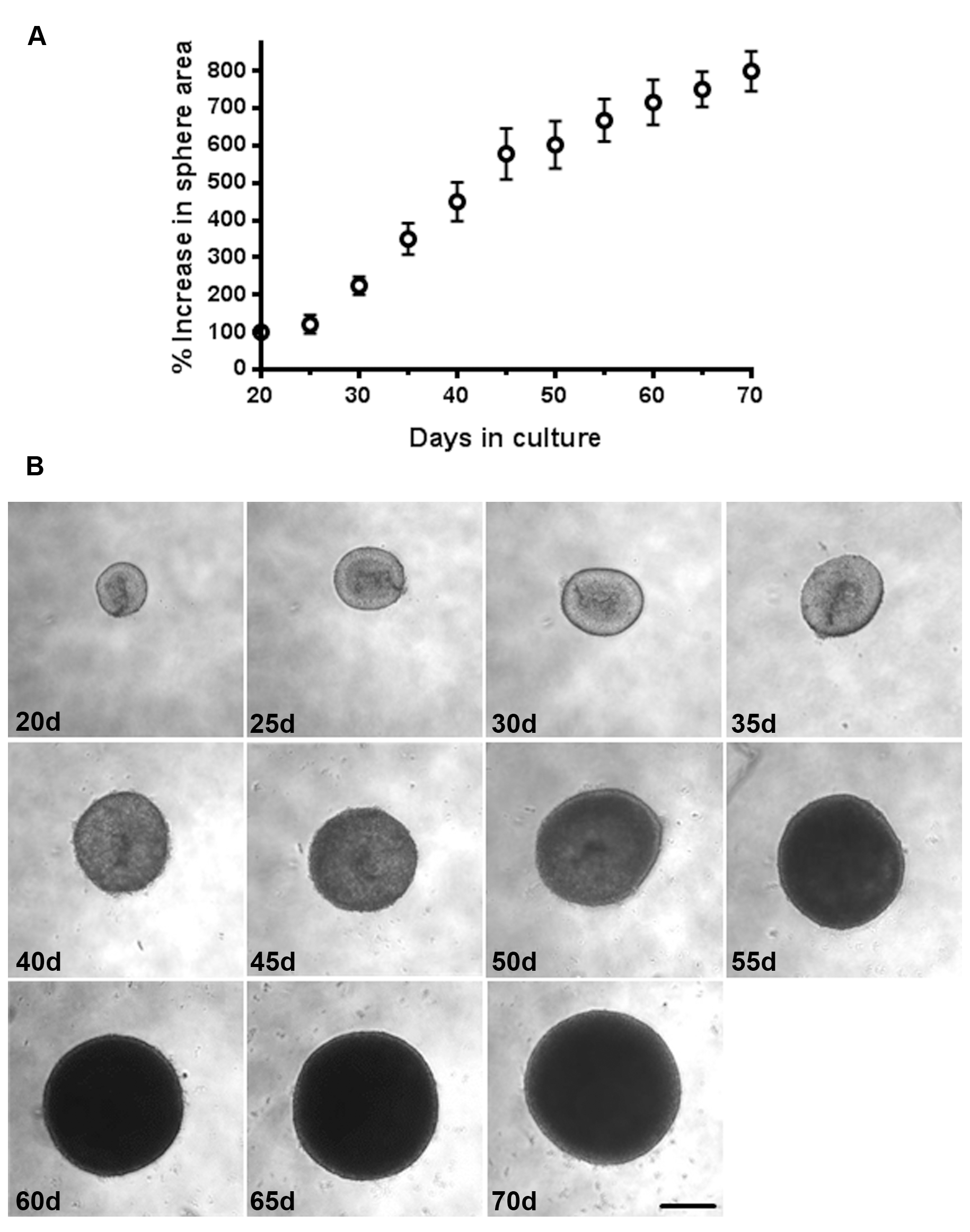

Supplement: S6 Fig — Individual day 20 hESC-OVs (n = 6) were isolated and grown for an additional 50 days in individual wells of a 96-well plate. (A) Phase-bright light microscopic images were taken every 5 days, followed by assessment of sphere area and percent increase in size over time. (B) Representative images of a single sphere over time. Scale bar: 100 μm. (TIF) [file pone.0135830.s006.tif]
