# Supplementary material for: VSX2 and ASCL1 Are Indicators of Neurogenic Competence in Human Retinal Progenitor Cultures
Source: PLoS One. 2015 Aug 20;10(8):e0135830. doi: 10.1371/journal.pone.0135830 (PMC4546156; doi:10.1371/journal.pone.0135830)
Supplement: S1 Table — (DOCX) [file pone.0135830.s007.docx]

| **Gene** | **Forward** | **Reverse** | **Size (bp)** |
| --- | --- | --- | --- |
| ß ACTIN | GCG AGA AGA TGA CCC AGA TC | CCA GTG GTA CGG CCA GAG G | 103 |
| ASCL1 | ACT GGG ACC TGA GTC AAT GC | TTA AGA AAG GGC GAG GAG GT | 160 |
| DLL1 | AGT GAG ATG GCA AGA CTC CCG TTT | TCT GAA CTC GGT TTC TCA GCA GCA | 122 |
| GAPDH | ACC ACA GTC CAT GCC ATC AC | TCC ACC ACC CTG TTG CTG TA | 450 |
| HES6 | AGC TAC GGG CAG GAG GAA GAA TTT | ACA CTA GTG CCC AGC ACC ATT TCT | 127 |
| VSX2 | ATT CAA CGA AGC CCA CTA CCC AGA | ATC CTT GGC TGA CTT GAG GAT GGA | 229 |
